# A Multidisciplinary Intervention to Reduce Infections of ESBL- and AmpC-Producing, Gram-Negative Bacteria at a University Hospital

**DOI:** 10.1371/journal.pone.0086457

**Published:** 2014-01-23

**Authors:** Jenny Dahl Knudsen, Stig Ejdrup Andersen, for the Bispebjerg Intervention Group

**Affiliations:** 1 Department of Clinical Microbiology, Copenhagen University Hospital, Hvidovre Hospital, Hvidovre, Denmark; 2 Infection Control Organisation, Copenhagen University Hospital, Bispebjerg Hospital, Copenhagen NV, Denmark; 3 Department of Clinical Pharmacology, Copenhagen University Hospital, Bispebjerg Hospital, Copenhagen NV, Denmark; University of North Dakota, United States of America

## Abstract

In response to a considerable increase in the infections caused by ESBL/AmpC-producing *Klebsiella pneumonia* in 2008, a multidisciplinary intervention, with a main focus on antimicrobial stewardship, was carried out at one university hospital. Four other hospitals were used as controls. Stringent guidelines for antimicrobial treatment and prophylaxis were disseminated throughout the intervention hospital; cephalosporins were restricted for prophylaxis use only, fluoroquinolones for empiric use in septic shock only, and carbapenems were selected for penicillin-allergic patients, infections due to ESBL/AmpC-producing and other resistant bacteria, in addition to their use in severe sepsis/septic shock. Piperacillin-tazobactam ± gentamicin was recommended for empiric treatments of most febrile conditions. The intervention also included education and guidance on infection control, as well as various other surveillances. Two year follow-up data on the incidence rates of patients with selected bacterial infections, outcomes, and antibiotic consumption were assessed, employing before-and-after analysis and segmented regression analysis of interrupted time series, using the other hospitals as controls. The intervention led to a sustained change in antimicrobial consumption, and the incidence of patients infected with ESBL-producing *K. pneumoniae* decreased significantly (*p*<0.001). The incidences of other hospital-associated infections also declined (*p’s*<0.02), but piperacillin-tazobactam-resistant *Pseudomonas aeruginosa* and *Enterococcus faecium* infections increased (*p*’s<0.033). In wards with high antimicrobial consumption, the patient gut carrier rate of ESBL-producing bacteria significantly decreased (*p* = 0.023). The unadjusted, all-cause 30-day mortality rates of *K. pneumoniae* and *E. coli* were unchanged over the four-year period, with similar results in all five hospitals. Although not statistically significant, the 30-day mortality rate of patients with ESBL-producing *K. pneumoniae* decreased, from 35% in 2008–2009, to 17% in 2010–2011. The two-year follow-up data indicated that this multidisciplinary intervention led to a statistically significant decrease in the incidence of ESBL/AmpC-resistant *K. pneumoniae* infections, as well as in the incidences of other typical hospital-associated bacterial infections.

## Introduction

Infections caused by extended-spectrum beta-lactamase (ESBL)-producing, Gram-negative bacteria are associated with increased morbidity and mortality, which is linked to inappropriate or delayed antimicrobial treatment. Other risk factors include severe co-morbidities, old age, longer hospital stays, intensive care unit stays, and previous treatment with antimicrobial agents [1]–[8]. Antibiotic selection of ESBL-producing, Gram-negative pathogens is related to both the total use of antimicrobial agents in departments or hospitals, but also to the particular use of the fluoroquinolones, and second and third generation cephalosporins [3]–[5], [7]–[9].

In 2008, the Department of Clinical Microbiology (DCM), serving five somatic hospitals in Copenhagen, Denmark, noticed a considerable increase in the ESBL/AmpC-producing bacterial infection rates at two hospitals. The outbreak was mainly due to *Klebsiella pneumoniae*, later shown to be of clonal origin [10], [11]. Reinforced infection control and surveillance measures proved to be insufficient. It was not possible to identify any source for these bacteria, as infections were seen in patients all over the hospitals, but especially among elderly patients. Some studies had reported on the benefit of decreasing broad-spectrum antibiotic use, e.g. with regard to a reduction in incidences of ESBL-producing bacterial infections [12]–[18]. These studies served as inspiration for us, in planning an intervention. All of these interventions however were single hospital studies, and only few have investigated the effect of antimicrobial restriction on mortality or prevalence of other nosocomial pathogens.

In the present study, we report the results of an intervention at the Copenhagen University, Bispebjerg Hospital (BBH), using four other hospitals as controls. The intervention started January 18, 2010. The investigated effect parameters included: antibiotic consumption, proportion of ESBL-producing *K. pneumoniae* and *Escherichia coli* in clinical samples, point prevalence of fecal carriage of ESBL-producing bacteria, which was conducted prior to and after intervention, incidence of ESBL-producing bacterial infections, as well as the incidence of infection *Staphylococcus aureus*, *Enterococcus sp*., *Pseudomonas aeruginosa*, *Clostridum difficile*, and others, and all-cause 30-day mortality in bacteraemia patients. The same parameters were also investigated in four non-intervention hospitals, for comparison controls.

## Materials and Methods

### Hospital Settings

The intervention took place at a university hospital, Bispebjerg Hospital (BBH). Before 2011, the Department of Clinical Microbiology (DCM), Hvidovre Hospital (HVH) served itself, BBH, and three other somatic hospitals: Frederiksberg Hospital (FBH), which also experienced high rates of ESBL-producing *K. pneumoniae* and *E. coli* infections, Amager Hospital (AMH), and Bornholms Hospital (BOH). Data from HVH, AMH, and FBH were used for comparison, but not from BOH, due to the hospital’s small size. For these hospitals, the awareness of antimicrobial guidance was reinforced through educational workshops at least once in the spring of 2010. Moreover, all physicians in the DCM routinely advised clinical doctors to avoid the use of cephalosporins when penicillins could be used, and to generally minimize the use of fluoroquinolones.

For further comparison, we included Glostrup Hospital (GLH), which had been served by the DCM at Herlev Hospital until January 2011, when the DCM at HVH took over the microbiological service. For key data, please see Table 1.

**Table 1:** Key data for the hospitals in 2010.

	*Type of departments*	*Number* *of beds*	*Number of* *occupied* *bed-days* *(OBD)*	*Number of* *discharges*	*Average* *length of stay*	*Number of* *ambulatory* *visits*	*Total consumption of* *J01 in DDD per 1000* *OBD*
**Bispebjerg Hospital (BBH)**	Acute medicine; ICU; Internal medicine wards for gerontology, cardiology, endocrinology, gastroenterology, neurology, rheumatology, and respiratory diseases; Orthopedic surgery; Gastrointestinal surgery, Dermato-venereology,Palliative therapy.	585	153,489	42,219	3.6	24,000	863
*Intervention hospital*							
**Amager Hospital (AMH)**	Acute medicine, Internal medicine wards for gerontology,cardiology, gastroenterology, and respiratory diseases;Orthopedic surgery	150	48,763	13,228	3.7	65,000	830
Control hospital for theintervention, served by thesame DCM							
**Hvidovre Hospital** **(HVH)**	Acute medicine, ICU, Pediatric department; Internal medicinewards for gerontology, cardiology, endocrinology, rheumatology,gastroenterology, and respiratory diseases; Orthopedic surgery,Gastrointestinal surgery, Obstetric-gynecology; Training center forposttraumatic disabilities	575	196,680	65,522	3.0	295,000	776
Control hospital for theintervention, served by thesame DCM							
**Frederiksberg Hospital (FBH)**	Acute medicine; ICU; Internal medicine wards for gerontology,rheumatology, cardiology, endocrinology, gastroenterology, andrespiratory diseases; Orthopedic surgery; Obstetric-gynecology(closed in 2009)	250	68,407	19,807	3.5	109,000	858
Control hospital for theintervention, served by thesame DCM							
**Glostrup Hospital (GLH)**	Acute medicine, ICU, Pediatric department (closed in 2009);Internal medicine ward, rheumatology, and neurology;Orthopedic surgery; Ophthalmology.	300	116,846	30,828	3.8	230,000	589
Control hospital for theintervention, served byanother DCM untilJanuary 2011.							

### The Project at Bispebjerg Hospital

The intervention included isolation precautions, communication, and education, in addition to antimicrobial stewardship and restrictions. A taskforce, which consisted of a clinical microbiologist, a clinical pharmacologist, a pharmacist, two infection-control nurses, and quality assessment (QA) staff, was established to lead four working-groups. The project’s steering committee comprised of the hospital managers, the clinical microbiologist, the clinical pharmacologist, and the head of the QA department. The strategy of the intervention was to involve most staff members, the theory behind which is described elsewhere [19].

#### Antimicrobial stewardship

All general and local guidelines on the use of antimicrobials for empirical treatments and prophylaxis were collected and revised, restricting cephalosporins entirely for prophylaxis only, and fluoroquinolones for septic shock. Before the intervention, cefuroxime ± gentamicin ± metronidazol were used as the primary drugs of choice, empirically for the most serious febrile conditions. After the start of the intervention, piperacillin-tazobactam ± gentamicin was recommended, including for the empirical treatment of fevers of unknown or undocumented causes. For patients who are allergic to penicillins, carbapenems were recommended, with ertapenem as a first-line treatment, while meropenem was recommended for documented or suspected infections of ESBL/AmpC-producing bacteria or *Pseudomonas sp*., and for septic shock.

The stewardship encouraged the principal prescription of narrow-spectrum penicillins when infections were microbiologically diagnosed. For example, intravenous dicloxacillin or oral flucloxacillin were recommended for methicillin-susceptible *Staphylococcus aureus*, while benzyl- or phenoxymethyl-penicillin were the antimicrobials of choice for pneumococci and other streptococci. Fluoroquinolones were still allowed, but the stewardship suggested that they should be avoided if alternatives could be used.

As before the intervention, cephalosporins were recommended for surgical prophylaxis: 1.5 g cefuroxime once before orthopaedic surgery (three doses on the day of alloplastic implantations), before pacemaker-implantations, and together with metronidazole for abdominal surgery. Ceftriaxone was still available in the emergency room for treatment of meningitis.

A new pocket pamphlet describing the general guidelines for sepsis, urinary tract infections, and pneumonia was printed and distributed to all physicians, in addition to its publication on the hospital internal electronic net (BBH-intranet). The revised, ward-specific guidelines were presented and discussed as the intervention was introduced through separate educational workshops in each department. At these sessions, emphasis was made on collecting specimen samples for general microbiological diagnostics, and especially before starting antimicrobial treatment. We did not demand preauthorization of antimicrobial prescriptions. The ward’s pharmacy assistants were instructed to increase the physician’s awareness of the new regimens, and to ensure that the guidelines were followed, if possible. Where cephalosporin prophylaxis was not needed, pharmacists removed all cephalosporins from the ward’s medicine cabinets.

#### Infection control

On all work-days, the infection-control nurses intensified the ward visits, and, when needed, they gave guidance to the ward staff, especially on isolation precautions and hand hygiene. The infection-control nurses were electronically notified about all exceptional microbiological findings, such as cases of ESBL-producing bacteria, *Clostridium difficile*, methicillin-resistant *S. aureus*, and multiple-drug-resistant *Acinetobacter* or *Pseudomonas sp*.

For 18 months, the head-nurses of each ward reported weekly all cases of isolation-precautions, together with all reasons for and the duration of the procedures, to the infection-control secretary. Most often isolation precautions implied one patient in a single room, but seldom two or three patients were cohort isolated for the same condition in the same room. All staffs use gowns and gloves entering rooms with patients in isolation precautions. Three other infection-control initiatives were launched five months after the start of the intervention to enhance awareness on isolation precautions and compliance to guidelines regarding this: 1) all electronic requisitions for an investigation or service from other departments were equipped with a mandatory prompt, notifying if the patient was in isolation precautions; 2) a sticker of a yellow triangular sign was used for marking the beds of isolated patients, and on the doors of rooms with isolated patients; and 3) an insert with an ESBL tick-box for the patients’ paper-record. A pamphlet was published that explained the isolation precautions to patients and relatives. A video describing how to perform a tracheal suction was made available on the BBH intranet. Additionally, an E-learning program was initiated on tracheal suction, with special references to nurses and junior doctors.

#### Swabbing for fecal carriage of ESBL/AmpC-producing Enterobacteriaceae

Twice before and twice after the intervention started, screening for fecal carriage of ESBL/AmpC-producing bacteria was performed in three different wards, using rectal swabs. Two wards with relatively high antimicrobial consumption were included, such as the ward for orthopedic infections and the department for chronic wounds, which mainly treats diabetic foot ulcers. As a reference, a ward for general orthopedic surgery was included, a ward with relatively low antimicrobial consumption, almost entirely for prophylactic use. All patients admitted on the day of surveillance were asked to participate, and less than 10% denied participation. The results of this screening were hidden from clinical samples in the laboratory information systems, in order not to influence treatment of the individual patients.

Immediately before the start of the intervention, a small pilot study was performed on the fecal carriage of ESBL/AmpC-producing bacteria in patients upon admission to the acute medical ward.

#### Communication and feedback

Before the start of the intervention, information was given separately to each department, the new pamphlet was distributed among all physicians, and samples were placed in all medicine cabinets. The project used a logo, and for two months the intervention was announced on the opening page of the BBH-intranet, with links to further information and contact addresses. For a year, links to a description of the intervention, as well as updated results, were presented on the opening site of the BBH-intranet. Four newsletters, which printed results of the study, were mailed to the heads of each department, and were also published on the BBH-intranet.

### Ethical Considerations

The intervention was performed as part of the surveillance, which is routinely performed by the DCM, the local Infection Control Organization, and the Department Of Clinical Pharmacology. The Regional Committee on Biomedical Research Ethics was consulted, and provided a statement that they had been notified and no further approvals were required (Journal no. H-C-FSP-2009/9). The Public Health Medical Office of the Danish National Board of Health and the Department of Epidemiology at Statens Serum Institut (Copenhagen, Denmark) were both consulted during planning of the intervention.

### The ESBL Investigations and Diagnosis

The laboratory screening for ESBL- or AmpC-producing microorganisms in samples from patients included the use of a cefpodoxime disk for all Gram-negative bacteria (Oxoid®, UK) on Mueller-Hinton agar (DCM, Herlev Hospital, Denmark), according to the EUCAST guidelines (http://www.eucast.org/clinical breakpoints/). The confirmatory tests were performed using the MAST DISCS ™ ID AmpC and ESBL Detection Discs (D68C, Mast Group Ltd, UK). Selected isolates were further investigated using various molecular methods [9], [10].

Fecal carrier sampling was performed using E-swabs (481C, Copan Diagnostics, Inc., USA), and cultivated on a selective agar for Gram-negative bacteria (Brom-thymol Blue plate, 694, Statens Serum Institut, Denmark) with a cefpodoxime disk (Oxoid®, Thermo Fisher, UK). Species identification, susceptibility testing, and production of ESBL and AmpC further characterized the bacterial growth within the disk zone.

### Data Sources

Data on the susceptibility to cefuroxime was retrieved from the clinical microbiological database and used for the surveillance given to the hospital employees as monthly feedback. Cefuroxime susceptibility was noted for all isolates identified in the DMC, but it was still used, despite the fact that these not only included the ESBL-producing isolates, but also the AmpC-positive and the TEM1-hyperproducing isolates (for details, please see Method S1).

Incidences of various bacterial and yeast species were surveyed and retrieved from the database in the DMC in order to determine any unintended effects of the intervention. The incidences of patients per 1000 occupied bed-days (OBD) per year infected with *S. aureus*, *Pseudomonas aeruginosa*, *Enterobacter cloacae*, *Candida sp*, enterococci, and *C. difficile*, were collected to study the effect of the changed antimicrobial consumption. Patients were included only once per year, per species.

All bacteraemia patient data were retrieved from the clinical microbiological database, and the 30-day mortality data following bacteraemia episodes were obtained from the daily updated Central National Register on vital status, using the unique personal number that all Danish citizens hold.

Data on consumption of antimicrobial agents were retrieved from the hospital pharmacy database as monthly data on the usage of all antibacterial agents, such as J01 [Anatomical-Therapeutic-Chemical (ATC) code J01 (http://www.whocc.no). Data on the monthly OBD were retrieved from the hospitals’ administrative systems.

### Statistical Analyses

From January 2008 through January 2012, monthly data were collected on the cefuroxime resistance of *E. coli* and *K. pneumoniae* separately, antibiotic usage, and the number of occupied bed-days. To adjust for the monthly variation in the number of patients treated, the monthly data on drug use (in defined daily doses (DDDs) (http://www.whocc.no) or cases of infection of ESBL/AmpC-producing bacteria were divided by the monthly OBD. Since the intervention was fully implemented on January 18^th^ 2010, data from this month were omitted from the analyses, leaving 24 points before, and 24 points after the start of the intervention for the analyses [20]. The effect size of the intervention on antibiotic use was assessed with before-and-after analysis and segmented regression analysis of interrupted time series (ITS) data [21]. For the latter, the break point was set to January 2010. Controlling for seasonality, sequential data from the intervention hospital were analyzed using the following modified model, adopted from Höegberg et al [22] (for detail, please see Methods S2).

For comparison of frequencies, the Chi-square test was used, unless the number of patients in one of the groups was below 50, where Fisher’s exact test was used (GraphPadPrism 5.04 GraphPad Software, Inc., California, USA). *P*-values less than 0.05 were considered statistically significant.

## Results

### Antimicrobial Consumption

**Figure 1:**
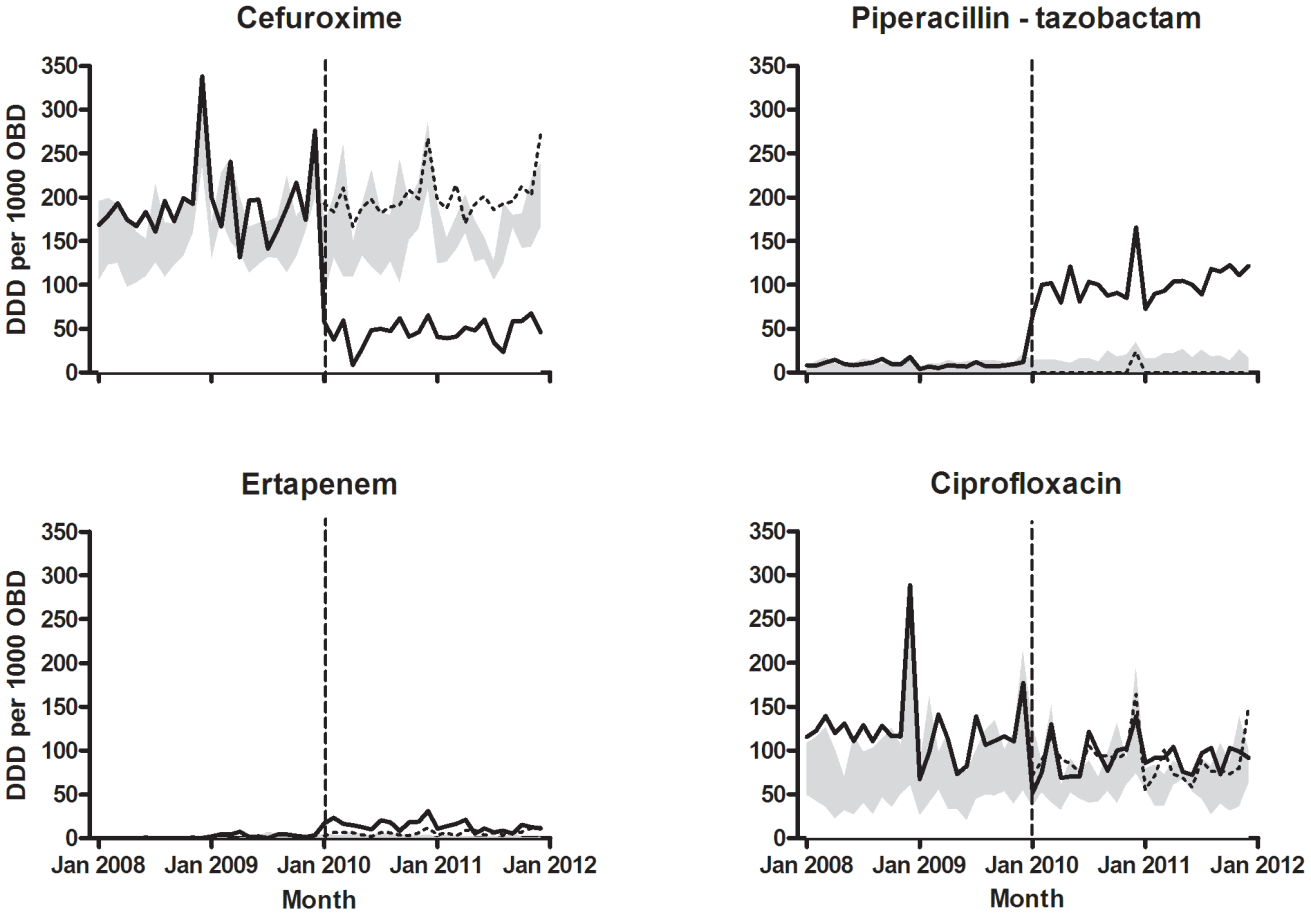
Monthly consumption of antimicrobials. Bold lines show the monthly consumption at the intervention hospital. Dashed lines represent the predicted usage. Grey shaded bands illustrate the range of the monthly use at the four control hospitals. DDD = defined daily doses; OBD = occupied bed-days. The consumption of ciprofloxacin did not changed significantly, but shifts in levels of consumptions of cefuroxime (*p*<0.001), ertapenem (*p* = 0.001), and piperacillin-tazobactam (*p*<0.001) at the intervention hospital were statistically significant, as indicated by segmented regression analysis of interrupted time series.

Coinciding with the launch of the new antimicrobial guidelines, the antimicrobial consumption changed rapidly and significantly, as shown in Figure 1. Hence, the before-and-after analyses at the intervention hospital indicated several changes in post-intervention mean use of antibiotics, which was most pronounced for cefuroxime, ertapenem, and piperacillin-tazobactam (Table 2). Moreover, this analysis indicated marginal increases in the mean use of ertapenem and the piperacillin-tazobactam combination at the control hospitals.

**Table 2:** Before-and-after analysis of drug use at the intervention and control hospitals.

*Antimicrobial agent*	*Department*	*Inter-cept*	*Slope*	*p-value for* *slope*	*Change in* *mean*	*p-value for* *change in* *mean*	*Change* *in slope*	*p-value for* *change in slope*
**Phenoxymethyl-penicillin and** **benzyl-penicillin**	Intervention	81.7	−0.7	0.23	35.8	0.005	−0.8	0.25
	Control	80.4	−0.1	0.65				
**Ampicillin and amoxicilin**	Intervention	162.0	−0.6	0.55	−15.2	0.43	0.0	1.00
	Control	102.7	0.1	0.68				
**Amoxicillin with inhibitor**	Intervention	48.7	0.1	0.82	4.8	0.71	−0.1	0.87
	Control	51.9	0.3	0.051				
**Piperacillin-tazobactam**	Intervention	1.9	−0.8	0.16	91.1	<0.0001	1.3	0.014
	Control	2.4	0.2	<0.0001				
**Dicloxacillin and flucloxacillin**	Intervention	58.2	−0.2	0.86	44.0	0.15	−1.3	0.052
	Control	33.3	0.3	0.04				
**Cefuroxime**	Intervention	185.6	0.3	0.74	−143.8	<0.0001	−0.7	0.52
	Control	145.4	−0.1	0.81				
**Ertapenem**	Intervention	−1.5	0.2	0.08	15.1	<0.0001	−0.5	0.0003
	Control	0.2	0.0	0.20				
**Meropenem**	Intervention	24.8	0.0	0.96	−2.5	0.70	−0.2	0.64
	Control	12.6	0.1	0.07				
**Ciprofloxacin**	Intervention	108.3	−1.5	0.08	−6.4	0.70	0.7	0.50
	Control	74.5	−0.4	0.002				
**Macrolide antibiotics**	Intervention	27.9	0.5	0.22	6.1	0.43	−0.2	0.65
	Control	18.6	0.2	0.02				

ITS analysis of the average monthly antibiotic consumption, given as defined daily doses (DDD) per 1000 occupied bed-days (OBD) (for more detail about the parameters, please see Methods S2).

The ITS analysis unveiled immediate effects of the intervention in terms of statistically significant shifts in the level of the post-intervention consumption of cefuroxime (−143.8 DDD per 1000 OBD per month, *p*<0.0001), ertapenem (+15.1 DDD per 1000 OBD per month, *p*<0.0001), beta-lactamase sensitive penicillins (phenoxy- and benzyl-penicillins) (+35.8 DDD per 1000 OBD per month, *p* = 0.005), and piperacillin-tazobactam (+91.1 DDD per 1000 OBD per month, p<0.0001). Only piperacillin-tazobactam had a different slope after the intervention. The intervention affected neither the slope, nor the mean consumption of amoxicillin with enzyme inhibitor, ciprofloxacin, meropenem, macrolide antibiotics, or the different penicillins.

Comparison of the average drug consumption at the four control hospitals indicated that the pattern described above was specific to the intervention hospital. Hence, the ITS analysis of data from the control hospitals revealed only a marginal decrease in the average consumption of ciprofloxacin, and increases in the average consumption of macrolide antimicrobials, beta-lactamase-resistant penicillins (dicloxacillin and flucloxacillin), and piperacillin-tazobactam. The average consumption of ciprofloxacin showed a decreasing trend (−0.4 DDD per 1000 OBD per month, *p* = 0.002), while the average consumption of macrolide antibiotics, betalactamase stable penicillins, and piperacillin-tazobactam showed a slightly increasing trend (Table 2). The consumption of amoxicillin-enzyme inhibitor, cefuroxime, ertapenem, meropenem, beta-lactamase-sensitive penicillin, and extended-spectrum penicillins remained unchanged.

The total consumption of antimicrobials at BBH increased during the intervention. Thus DDD per 1000 OBD in 2008, 2009, 2010, and 2011 were 739, 770, 896, and 849, respectively. This cannot be explained by the shift from cefuroxime to piperacillin-tazobactam, because piperacillin(tazobactam) 4(0.5) g t.i.d. represents 0.86 DDD while the former used cefuroxime 1.5 g t.i.d represents 1.5 DDD.

### Impact on ESBL-producing Bacteria

**Figure 2:**
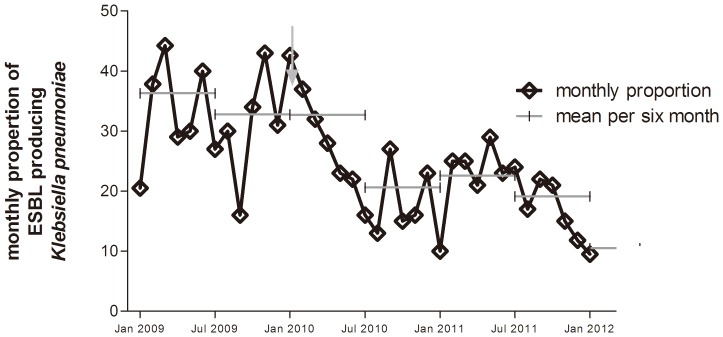
The monthly proportions of ESBL/AmpC-producing isolates of *K. pneumoniae* in patient clinical samples at Bispebjerg Hospital. The average proportion per six months is shown as grey lines. The arrow indicates the start of the intervention.

**Figure 3:**
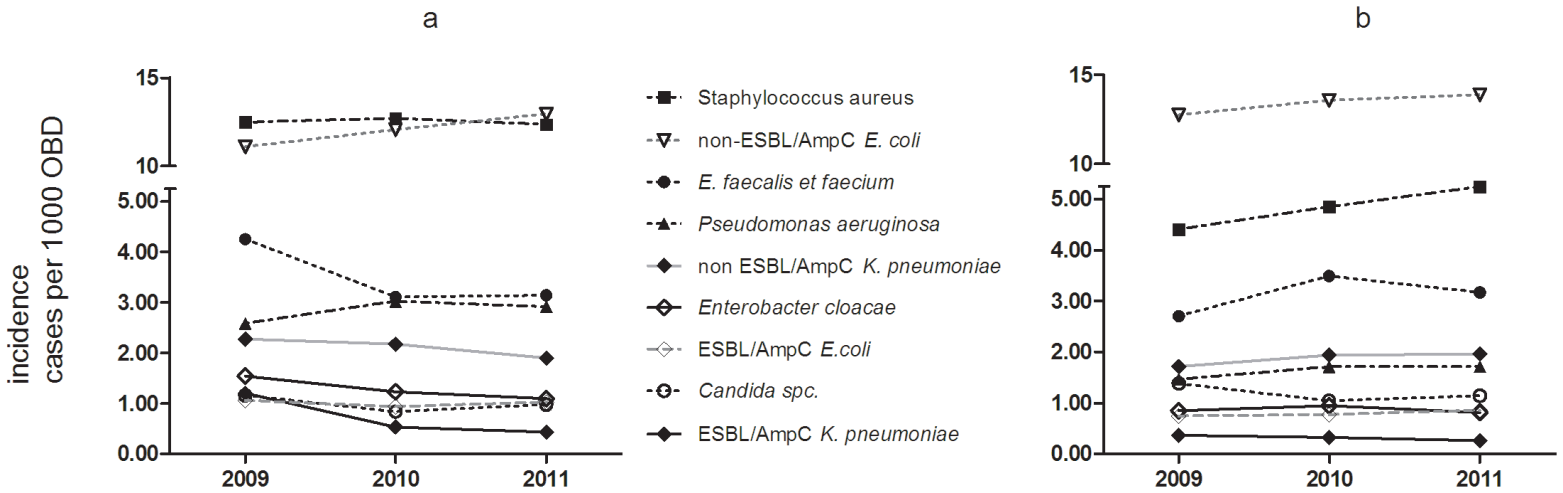
Annual incidences of patients infected with various bacterial species. The annual incidences of patients, as number per 1000 occupied bed days (OBD), infected with various bacterial species at (a) Bispebjerg Hospital and (b) the four control hospitals. At Bispebjerg Hospital (a), the incidence of infections decreased for ESBL/AmpC producing *K. pneumoniae* (*p*<0.001), *Enterococcus sp*. (*p*<0.0001), *E. cloacae* (*p = *0.002), and *Candida sp*. (*p* = 0.018). The Incidences of infections with non-ESBL/AmpC *K. pneumonia,* ESBL/AmpC producing *E. coli, S. aureus* and *P. aeruginosa* were unchanged (*p*’s>0.063), and the incidence of infections increased for non-ESBL/AmpC producing *E. coli* (*p*<0.001). At the other hospitals (b) the incidence of infections with *S. aureus*, and *Enterococus sp*. increased significantly (*p’s*<0.015), the incidences of infections with *Candida sp, ESBL/AmpC producing E. coli, non-ESBL/AmpC producing E.coli, P. aeruginosa, E. cloacae*, were unchanged (*p’*s>0.06).

**Figure 4:**
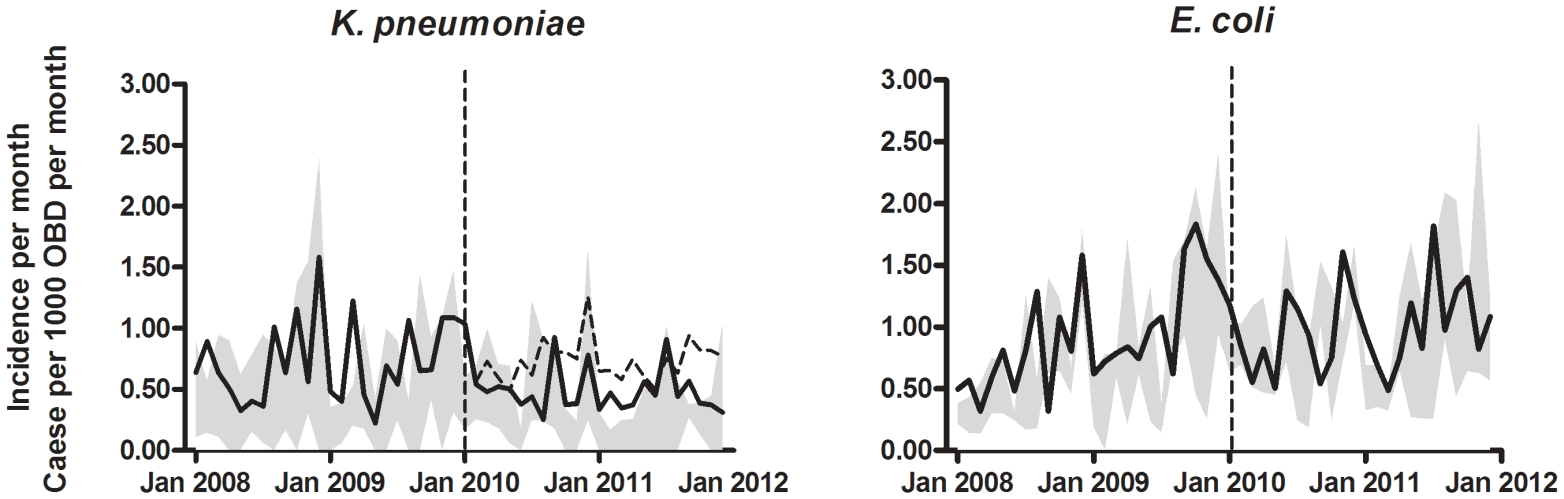
Annual incidences of patients infected with ESBL/AmpC-producing bacteria. The number of new patients with ESBL- or AmpC- producing *K. pneumoniae* (upper panel) and *E. coli* (lower panel) per 1000 occupied bed days (OBD) per month. The dashed line represents the predicted incidence given that the intervention had not taken place. Grey shaded bands illustrate the range at the four control hospitals.

A statistically significant effect of the intervention was seen on the monthly proportion (Figure 2), and the incidence of patients with ESBL-producing *K. pneumoniae* infections (Figure 3 and 4) (*p’s*<0.001), while the effect on incidence of ESBL-producing *E. coli* infections was insignificant (*p* = 0.524) (Figures 3 and 4). The most pronounced effect was observed in the orthopedic surgery wards where the ESBL/AmpC-producing bacterial infection proportions decreased from a baseline value of 51% before the intervention in 2009, to 23% in 2010, and 26% in 2011 (*p* = 0.0011). Likewise, in the geriatric ward, a marked decrease from 50% in 2009, to 13% and 22% in 2010 and 2011, respectively, was observed (*p* = 0.0332). For male patients, the proportions of ESBL and AmpC production in the *K. pneumoniae* isolates were 40%, 24%, and 22% in 2009, 2010, and 2010, respectively (*p* = 0.0001). For female patients, the proportions were 31%, 17% and 16%, respectively (*p*<0.0001). While no effect was seen in patients <60 years of age (*p* = 0.0953), a significant effect was observed for patients aged ≥60 years (36% in 2009, 21% in 2010, and 17% in 2011, *p*<0.001).

Figure 4 shows the actual and the expected numbers of patients at the intervention hospital with ESBL-producing *K. pneumoniae* or *E. coli.* For comparison, the actual number of patients at the four control hospitals is shown. A segmented regression analysis of interrupted time series (2008–2011) was conducted. A model with two break points best fitted the data on *K. pneumoniae*, and revealed two significant changes in trends at BBH, but not the other hospitals, separately or all together: the first was in January 2010 (from 0.001 to −0.110 patients per 1000 OBD per month, *p* = 0.015), and the second was in August 2010 (from −0.110 to −0.001 patients per 1000 OBD per month, *p* = 0.027). No significant shift in level was detected. In contrast, a model with no break points best fitted the data on *E. coli*, and suggested no effect of the intervention, although a borderline-significant, positive trend of 0.0068 (*p* = 0.071) was detected, indicating a possible increase in the incidence of ESBL-producing *E. coli*.

The incidence of both fully susceptible and resistant *K. pneumoniae*, as diagnosed findings in clinical samples per 1000 OBD, both declined, but the decline was only statistically significant for resistant *K. pneumoniae* (*p*<0.001) (Figure 3). The incidence of patients infected with the non-ESBL/AmpC-producing *E. coli* increased significantly (*p*<0.001), and the rate of ESBL/AmpC production in cases of *E. coli* decreased, although this result was not statistically significant (*p* = 0.148). The increasing incidence of the non-ESBL/AmpC-producing *E. coli* was observed almost entirely in urine samples from the acute medical ward (*p*<0.0001).

### Impact on Other Bacterial Species Found in Clinical Samples

**Figure 5:**
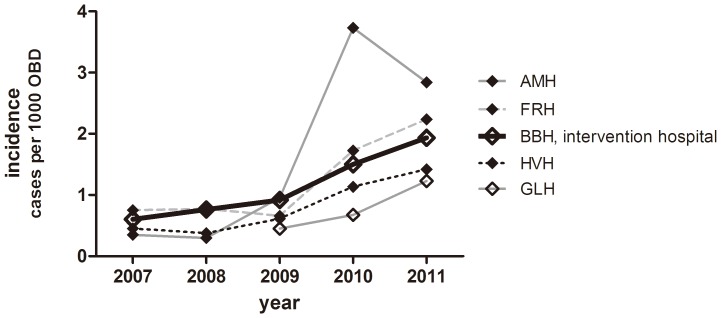
Annual incidences of patients infected with *C. difficile*. The annual incidence of patients with *Clostridium difficile* associated diarrhea, as number per 1000 occupied bed days (OBD), per year, at the different hospitals.

The incidence of patients at BBH with infections with various bacteria, such as enterococci, *E. cloacae* and *Candida* species, decreased significantly, (*p’s*<0.02). However, for *Enterococcus faecium* alone, although there were only a small number of cases, an increase was observed, *p* = 0.0142, (71 cases in 2009, 61 in 2010, 97 cases in 2011). At BBH, the incidence of *S. aureus* was unchanged. For patients in the other hospitals, the incidence of enterococci and *S. aureus* infections increased significantly (*p’s*<0.015), *Candida sp*., *Enterobacter cloacae, E. coli*, and non-ESBL-producing *K. pneumoniae* infections were unchanged. The incidence of *P. aeruginosa* infections was unchanged in all hospitals, but the number of piperacillin-tazobactam-resistant *P. aeruginosa* increased at BBH, albeit in small numbers, (17 patients in 2009, 22 in 2010, and 35 in 2011, *p* = 0.033) (Figure 3). We did not see any cases of piperacillin-tazobactam resistant *P. aeruginosa* bacteraemia. At all six hospitals, the incidence of *C. difficile* increased, which was most pronounced at one of the control hospitals (Figure 5).

### Carrier Studies

The fecal carrier studies in the two high and one low consumption wards prior to and two months after the start of the intervention, showed a significant decrease (*p* 0.0025) in ESBL carrier status in the high consumption wards from 31% to 10%, but no differences were found in the general orthopedic ward with low antimicrobial consumption (*p* = 1.00) (Table 3). Unfortunately, only 59 randomly selected patients could be enrolled into the carrier study of patients admitted to the acute medical ward prior to the intervention. Nine patients (15%; 95% CI: 8–27%) were carriers of ESBL- or AmpC-producing *E. coli*, while none carried ESBL- or AmpC-producing *K. pneumoniae.* Eight of the nine carriers had been hospitalized for at least two days within the last 12 months.

**Table 3:** Fecal carrier studies in three wards prior to and after start of the intervention.

	Before the intervention				After start of the intervention			
	First carrier study		Second carrier study	Sum	Third carrier study	Fourth carrier study		Sum
	No. of patients;No. positive (%)		No. of patients;No. positive (%)	No. of patients; No. positive(%, C.I.#)	No. of patients;No. positive (%)	No. of patients;No. positive (%)		No. of patients; No. positive(%, C.I.#)
**General orthopedic surgery** (low antimicrobial consumption)	16; 0 (0%)		17; 1 (6%)	33; 1 (3%; 0.5–15%)	10; 1 (10%)	7; 0 (0%)		17; 1 (6%; 1–27%)
**Orthopedic surgery specialized in infections** (high antimicrobial consumption)	16; 4 (25%)		18; 7 (39%)	34; 11 (32%; 19–49%)	14; 3 (21%)	10; 0 (0%)		24; 3 (13%; 4–31%)
**Center for chronic wounds** (high antimicrobial consumption)	8; 3 (38%)		10; 2 (20%)	18; 5 (28%; 13–51%)	5; 0 (0%)	10; 1 (10%)		15; 1 (7%; 1–30%)
Total number of patients	40; 7 (18%)		35; 10 (29%)	75; 17 (49%; 33–64%)	29; 4 (14%)	27; 1 (4%)		56; 5 (9%; 4–19%)
**Positive patients in low consumption department (%)**				**1 of 33 (3%**; 0.5–15%**)**				1 of 17 (6%; 1–27%)*
**Positive patients in highconsumption departments (%)**				**16 of 52 (31%; 20–44%)**				4 of 39 (10%; 4–24%)**
No. of patients with ESBL/AmpC-producing *E. coli*	3	5		8	2		0	2
No. of patients with ESBL-producing *Klebsiella pneumoniae*	4	3		7	1		1	2
No. of patients with ESBL-producing *Klebsiella ornithiolycica*	0	2		2	0		0	0
No. of patients with ESBL-producing *Proteus mirabilis*	0	0		0	1		0	1

The result of the 141 patients examined in four carrier studies and the findings of ESBL- or AmpC-producing Gram-negative bacteria.

# C.I.: 95% Confidence interval. Difference between findings before or after start of the intervention using Fisher exact test: *p  = 1.00; **p  = 0.0225.

### Bacteraemia

The unadjusted, all-cause 30-day mortality rates following bacteraemia was unchanged for the previous two years before the intervention, compared to two years after. These results were not only for the most common cases of *E. coli, S. aureus*, enterococci, pneumococci, non-hemolytic streptococci, *K. pneumoniae*, *Proteus mirabilis,* and *P. aeruginosa*, but also for all bacteraemic patients at the intervention hospital as a whole (data not shown). The unadjusted, all-cause 30-day mortality rates were similar at all five hospitals. However, although not statistically significant, the 30-day mortality rate of patients with ESBL-producing *K. pneumoniae* decreased from 35% (11 of 34 patients died) in 2008–2008, to 17% (5 of 29) in 2010–2011 (*p* = 0.25).

## Discussion

For the first eight months after the start of the intervention, we observed a steady decline in the monthly reported number of isolates of *K. pneumonia* in the intervention hospital. These results suggest that this multidisciplinary intervention and antimicrobial stewardship resulted in a significant decrease in the incidence of patients infected with ESBL-producing *K. pneumonia*, as the incidence did not decline in the other hospitals. As expected, no effect on the prevalence of patients with ESBL/AmpC-producing *E. coli* was seen, presumably because *E. coli* originates more from community rather than from hospital settings and it may also be a matter of transmissibility in hospitals. This finding is consistent with similar results from studies on changes in antimicrobial prescribing [12]–[18], [23].

While the decrease in cephalosporin consumption was rapid and in complete concordance with the new guidelines, the decrease in ciprofloxacin consumption was less than intended. Unfortunately, an epidemic of *C. difficile* infections, primarily of the strain, CD027, occurred in the Copenhagen area at the same time as this study, and affected all of the hospitals. Thus, despite the reduced consumption of cephalosporins, the increase in *C. difficile*-associated diarrhea was not prevented; this increase may have been supported, in part, by the undermined reduction in ciprofloxacin consumption [20]. Nevertheless, ciprofloxacin consumption did steadily decrease over time in all the hospitals.

The increase in the total antimicrobial consumption at BBH, was in concordance with increases seen at the other hospital included in our study, as also seen in the Danish nation average of antimicrobial consumption per OBD [24]. This can maybe be explained in more patients treated in less time, as the values for OBD steadily decrease over years [24].

The 30-day mortality rate after bacteraemia did not increase after the intervention, which could be expected from changing the empiric treatment from cefuroxime to piperacillin-tazobactam, as piperacillin-tazobactam is not recommended for treatment of *S. aureus* bacteraemia. However, in contrast to cefuroxime, piperacilin-tazobactam has been shown to be effective against ESBL-producing Gram-negative bacterial infections [25], [26]. Another positive effect of the change in antimicrobial usage was the significant decrease in typical hospital-associated bacterial infections, such as enterococci, *Candida* sp, and *E. cloacae*. In contrast, the increase in piperacillin-tazobactam-resistant *P. aeruginosa* and *E. faecium* infections was a drawback of the intervention, although the actual numbers of infections remained small. The finding of an increased number of patients with susceptible *E. coli* isolates, especially in urine samples from the acute medical patients, although the admission frequency was stable, was another interesting result of the intervention and may be due to the encouraged sampling in this ward.

Though it is difficult to single out one part of this multifactorial intervention as the most important, we believe that the antimicrobial stewardship played the greatest role, since the decrease in the proportion of resistant *K. pneumoniae* followed immediately after the start of the intervention. A recent review of the documentation of various infection-control measures concluded that no studies have been able to document an effect of these efforts in non-outbreak settings, but there have not been any studies, which were properly designed to address these issues [27]. Notwithstanding, results from the Israeli nation-wide intervention against carbapenem-resistant *K. pneumoniae* indicated that the institutions that were compliant to the rules for isolation-precautions were more successful in decreasing the number of infected patients [28]. Likewise, reinforced infection-control measures were regarded most important in the management of an outbreak of ESBL-producing *K. pneumoniae* in a Belgian intensive care unit [29].

In the Israeli nation-wide intervention on carbapenem-resistant *K. pneumoniae*, an immediate decrease in the incidence was reported to little less than half of the maximum during the outbreak, and though a further steady decrease followed for at least one year, the incidence did not reach the pre-outbreak level [28]. Similarly, we saw an initial significant effect in our study, while a steady, but less pronounced, decrease continued thereafter.

Other investigators have reported decreases in frequencies of ESBL-producing, Gram-negative bacteria from hospitals or wards by changing antimicrobial consumption from cephalosporins to penicillins [12]–[17], [23]. Recently, a Swedish study that replaced penicillins for cephalosporins in a setting not much different from ours, succeeded in the eradication of a hospital outbreak of ESBL-producing *K. pneumoniae* [23]. In the Swedish study, the epidemic strain was eradicated from the hospital, something that has yet to occur in our intervention hospital.

Our carrier studies revealed a large variation in fecal ESBL-producing bacterial carriage rates among patients. Thus, only 1 of 40 patients were carriers in the general orthopedic surgery wards with low antimicrobial consumption (∼3–6%), while up to 11 of 32 patients (32%) carried ESBL-producing bacteria in the specialized ward for orthopedic surgical infections. The carrier prevalence for patients at admission to the medical ward was 15%, but this was almost entirely correlated to prior hospitalization. We concluded, although our data are sparse, that the carrier rates reflect the medical history for the patients. Carrier rates, studied as a point prevalence study in Israel during the national outbreak of carbapenem-resistant *K. pneumoniae*, also varied among patient groups (8–9% among medical and surgical patients; none in the pediatric ward) [30].

Segmented regression analysis of interrupted time series (ITS) is a modeling technique that allows control for prior trends in the drug use pattern, and displays the dynamics of response to an intervention [21]. Considered the strongest quasi-experimental test for analyzing the longitudinal effects of complex interventions at the institutional level, where a randomized, controlled trial is not feasible, this approach allows for the assessment of immediate and sustained changes of drug use in response to the new recommendations for antibiotic use, which were implemented at the intervention hospital. Moreover, data were collected at four control hospitals in the same region where no intervention had taken place. The ITS analysis displayed an immediate and sustained, statistically significant change in antimicrobial consumption that was not seen at the control hospitals; the consumption of cefuroxime was not expected to reduce to zero, since it was still used for surgical prophylaxis. Surprisingly, no significant shift in level of ciprofloxacin usage was seen, and the decreasing trend from a very high baseline consumption at pre-intervention was unaffected by the intervention. The ciprofloxacin consumption at the intervention hospital exceeded the level at the control hospitals over nearly the entire four-year observation period,

The ITS analysis of the incidence of ESBL-producing *K. pneumoniae* documented that the two significant changes in trend sub-testing of a shift from a higher to a lower level had taken place from January to August 2010. These data suggest that the intervention was successful in decreasing the incidence of patients suffering from ESBL/AmpC-producing *K. pneumonia* infections, and, in addition, it alerted the hospital organizations to being aware of and prepared for upcoming new infectious emergencies.

Foodborne outbreaks with ESBL-producing *E. coli* are common, and food is presumed to be the most probable source for the epidemic spread of *E. coli* [3]. ESBL-producing *K. pneumoniae* have mainly been described in local hospital outbreaks, although occasionally described as having a food borne origin [31]. A recent, large outbreak in Europe, mainly in Germany, of a Shiga-toxin-producing *E. coli* strain, which also produced ESBL, resulted in hemorrhagic uremic syndrome in both adults and children, and was found to be related to fenugreek seed from Egypt [32]. Travel, especially to Asia or the Middle East, has been mentioned as a risk factor for fecal carriership of ESBL-producing bacteria [33]–[35]. Production and wildlife animals can act as reservoirs for ESBL-producing, Gram-negative bacteria, as well, functioning as vectors for spreading resistant bacteria around the globe [36].

The strengths of this study are the prospective collection and presentation of detailed data, the relatively long follow-up period, the inclusion of mortality estimates, the parallel study of changes in incidences of infections with other important bacterial pathogens, and the analysis using interrupted time series with segmented regression, which is considered the method of choice for the analysis of before-and-after, quasi-experimental study designs of effect. We could thereby document the impact of the intervention, which changed consumption of antimicrobials, on the incidence of patients infected with ESBL/AmpC-producing *K. pneumoniae*. The most important limitation to this study was that it was not a randomized, controlled trial. A reintroduction of cephalosporins in the study hospital could be a way to exclude the phenomenon “regression to the mean”, and to demonstrate their ability to select for ESBL-producing bacteria, but such a study is impossible for ethical reasons. We also acknowledge, that our intervention cannot in total, be transformed to other settings, but it can hopefully be an inspiration for others dealing with similar problems.

In conclusion, our multidisciplinary intervention, including antimicrobial stewardship, succeeded in causing a stable statistically significant decrease in the incidence of ESBL/AmpC-producing *K. pneumonia* infections. We also experienced a decrease in other hospitals associated infection, although we saw an increase in piperacillin-tazobactam resistant *P. aeruginosa* and *E. faecium* infections. Furthermore, it confirmed earlier evidence that suggested that the reduction in cephalosporin consumption, despite a parallel increase in use of broad-spectrum penicillins, leads to reduced selection of ESBL/AmpC-producing Gram-negative bacteria.

## Supporting Information

Methods S1 The errors due to use Cefuroxime susceptibility a proxy for ESBL-producing.
(DOCX)

Methods S2 Controlling for seasonality in the sequential data.
(DOCX)
