# Supplementary material for: A Multidisciplinary Intervention to Reduce Infections of ESBL- and AmpC-Producing, Gram-Negative Bacteria at a University Hospital
Source: PLoS One. 2014 Jan 23;9(1):e86457. doi: 10.1371/journal.pone.0086457 (PMC3900527; doi:10.1371/journal.pone.0086457)
Supplement: Methods S1 — The errors due to use Cefuroxime susceptibility a proxy for ESBL-producing. (DOCX) [file pone.0086457.s001.docx]

**Supporting information**

**Methods S1 The errors due to use Cefuroxime susceptibility a proxy for ESBL-producing.**

The errors due to use susceptibility to cefuroxime a proxy for ESBL-production in bacteria were considered minor. For example, in 2009, among 2687 *E. coli* isolates from BBH, 292 (10.9%) were cefuroxime resistant, and of these, only six were cefpodoxime susceptible (~0.03% of all, and 2.1% of the cefuroxime-resistant isolates). Therefore, these isolates were presumably only AmpC-positive, six of which were discovered to be phenotypically hyperproducing isolates (~0.03% of all isolates, and 2.1% of the cefuroxime-resistant isolates). Similar results were obtained for *K. pneumoniae* isolates from BBH: among 641 isolates, 231 (36.0%) were cefuroxime resistant; of these, four were susceptible to cefpodoxime (~0.6% of all, and 1.7% of the cefuroxime-resistant isolates), and three were hyperproducing isolates (~0.5% of all isolates, and 1.3% of the cefuroxime-resistant isolates). This error was of similar size at FBH, in 2009: among 1947 *E. coli* isolates, 202 were cefuroxime resistant (10.4%), 11 of these were susceptible to cefpodoxime (~0.6% of all, and 5.4% of the cefuroxime-resistant isolates), while none were hyperproducing isolates. Among 641 *K. pneumoniae* isolates, 231 (36.6%) were cefuroxime resistant, all of which were also resistant to cefpodoxime, thus, none were AmpC-producing alone or hyperproducing isolates.
