# Supplementary material for: A Multidisciplinary Intervention to Reduce Infections of ESBL- and AmpC-Producing, Gram-Negative Bacteria at a University Hospital
Source: PLoS One. 2014 Jan 23;9(1):e86457. doi: 10.1371/journal.pone.0086457 (PMC3900527; doi:10.1371/journal.pone.0086457)
Supplement: Methods S2 — Controlling for seasonality in the sequential data. (DOCX) [file pone.0086457.s002.docx]

**Supporting information**

**Methods S2 Controlling for seasonality in the sequential data.**

With the intension to control for seasonality, sequential data from the intervention hospital were analyzed using the following modified model, adopted from Höegberg et al [15]

$y_{t}$=β_0_ + β_1_ • T_t_ + β_2_ • D_t_ + β_3_ • P_t_ + β_t_ • Month_(i-2)t_ + ε_t_

The model was fitted using least squares regression. In the model, $y_{t}$is the DDD antibiotic use per 1000 OBD at time t, T_t_ is the time in months from the start of the observation period (January 2008), D_t_ is a dummy variable for pre (D_t_ = 0) or post intervention (D_t_ = 1), and P_t_ is time in months since the intervention. For t before the intervention, P_t_ is coded as 0. January was chosen as the reference month, while the remaining months represented dummy variables (Month_2_ = February, Month_3_ = March etc.). The coefficient β_0_ is the baseline use of antibiotics, β_1_ is the slope (trend) prior to the intervention, and β_2_ and β_3_ represent the change in level immediately after the intervention and the change in slope after the intervention, respectively. If the value of β_1_ was significantly different from 0, the pre-intervention slope was considered statistically significant. Likewise, if the values of β_2_ and β_3_ were significantly different from 0, they were interpreted as a change in the level and/or slope.

Data from the control hospitals (with no obvious break-point) were analyzed using the following model:

$y_{t}$=β_0_ + β_1_ • T_t_ + β_t_ • Month_(i-2)t_ + ε_t_

Twelfth-order autoregressive processes were chosen. Removing the least significant parameters first, all non-significant autoregressive parameters were removed from the models.

For the before-and-after analysis, the Mann-Whitney U test was used. All analyses used a two-tailed α level of 0.05, and were performed using JMP 8.0 with add-ins. By providing graphical user interfaces for selected SAS procedures, such as the AUTOREG procedure, these add-ins call SAS 9.1 (SAS Institute inc. Cary, NC, USA) to perform the analyses, and display the results in a JMP window.
